# Gut microbial metabolite connections to cardiovascular disease call for gutsy therapeutic approaches

**DOI:** 10.1172/JCI201468

**Published:** 2025-12-01

**Authors:** Frank Ruschitzka, Antonio Vidal-Puig, Seyed Soheil Saeedi Saravi

**Affiliations:** 1 Center for Translational and Experimental Cardiology, Department of Cardiology, University Hospital Zurich, University of Zurich, Zurich, Switzerland.; 2 University Heart Center, Department of Cardiology, University Hospital Zurich, Zurich, Switzerland.; 3 TVP Lab, WT/MRC Institute of Metabolic Science, University of Cambridge, Cambridge, United Kingdom.; 4 Centro de Investigacion Principe Felipe, Valencia, Spain.

Cardiovascular diseases (CVDs) currently account for over 40% of mortality in aging populations worldwide (1). Despite aggressive management of traditional cardiovascular and cardiometabolic risk factors such as LDL cholesterol, hypertension, and diabetes and despite advances in gerotherapeutics, including senolytics, mTOR inhibitors, AMPK activators, and NAD^+^ boosters, a powerful and modifiable determinant of cardiovascular aging remains underappreciated: the gut microbiota.

Throughout the twentieth century, scattered observations suggested that gut microbes shape host physiology, but the complexity and dynamic nature of microbial communities have historically eclipsed the tools available to interrogate them. The advent of high-throughput microbiome sequencing, coupled with untargeted metabolomics, in the early twenty-first century transformed the field and established the gut microbiota as a key regulator of human health (2). Moving beyond documenting association and pursuing more mechanistic investigations of microbe-host physiology will require coupling integrative functional studies that connect microbial metabolites to host biology, with translational trials testing whether targeting microbial functions can prevent or ameliorate CVD in aging. Here, we summarize mechanistic evidence linking microbial metabolites to CVD risk, examine their validity as early biomarkers, and describe interventional approaches aimed at modifying microbial function to improve cardiovascular and cardiometabolic health with the aim of motivating collaboration between microbiome and cardiovascular research efforts.

## Gut microbial metabolites modify CVD risk

Beginning in the 1970s and 1980s, changes in gut microbial composition were associated with traditional cardiovascular risk factors, including obesity, insulin resistance, and dyslipidemia. By the early 2000s, studies demonstrating resistance to diet-induced obesity in germ-free mice provided clear evidence that gut microbiota actively shape host metabolism. Early 16S-rRNA sequencing by Koren et al. suggested a potential link between gut microbiota and atherosclerosis, with residual bacterial DNA detected in human atherosclerotic plaques (3), yet these reports did not make direct associations with CVD. The gap was bridged between 2011 and 2013, when Stanley Hazen and colleagues demonstrated that microbial conversion of certain dietary nutrients, including lecithin, choline, and carnitine, into trimethylamine *N*-oxide (TMAO) plays a direct role in promoting atherosclerosis and predicts major adverse cardiovascular events in humans (4, 5). These foundational studies established that microbial function and metabolites, not just microbial presence, drive disease risk. Additional metabolites derived from amino acids abundant in Western diets, including phenylacetic acid (PAA), its derivative phenylacetylglutamine (PAGln), imidazole propionate (ImP), and *p*-cresol sulfate, contribute to atherosclerosis, heart failure (HF), and thrombotic risk.

Building on these findings, in 2025, our group (Saeedi Saravi et al.) (6) and Yang et al. (7) published parallel studies in *Nature Aging* revealing a mechanistic role of phenylalanine-derived metabolites, PAA and PAGln, in driving vascular endothelial senescence. Complementing these findings, recent large-scale proteomic blueprints of human organs have positioned vascular decline as both a sensor and driver of aging and age-related pathologies (8). Thus, microbial metabolites have emerged as not only early predictors but also modulators of pro-aging mechanisms in the vasculature, as discussed previously (9). Indeed, Wang and colleagues revealed divergent age- and metabolism-associated microbiota signatures that counteract increased CVD risk in metabolically unhealthy older adults (10), introducing the concept of a “gut microbial age” metric predictive of late-life CVD risk. This concept establishes a framework for future work integrating microbial signatures with complex metabolic phenotypes to refine CVD risk prediction in aging populations.

## Microbial metabolites emerge as predictive biomarkers in CVD

As sentinels of modern aging, microbial metabolites transduce environmental inputs into biological signals that shape cardiovascular decline or resilience. Although over half of circulating small molecules originate from gut microbes, few have been robustly implicated in major cardiometabolic diseases, including atherosclerosis and HF. Among them, circulating TMAO predicts long-term (~11-year) cardiovascular morbidity and mortality in diverse, multiethnic aged populations with atherosclerotic CVD (ASCVD) or acute coronary syndrome, independent of the traditional risk factor, hypercholesterolemia (11). Beyond ASCVD, TMAO also contributes to a 3.4-fold increased mortality risk and predicts 5-year in-hospital mortality in stable HF patients (12). Our group further reported that circulating TMAO increases with age and heightens thrombotic and mortality risks in older patients with atrial fibrillation (13, 14). Moreover, *N*,*N*,*N*-trimethyl-5-aminovaleric acid (TMAVA) derived from gut microbial metabolism of trimethyllysine — a TMA precursor — consistently predicts cardiac death and heart transplantation risk, independently of TMAO, over a 7-year follow-up in patients with HF (15). At the cellular level, elevated TMAO links to senescence hallmarks, including proinflammatory secretory reprogramming, oxidative stress, DNA damage (through the p53/p21^WAF1/Cip1^/Rb axis), and mitochondrial dysfunction. Mechanistically, TMAO promotes endothelial and vascular smooth muscle cell senescence through ROS-dependent p38-MAPK/NF-κB activation, triggering senescence-associated secretory phenotype (SASP) components such as IL-6 and TNF-α, which sustain chronic low-grade inflammation (inflammaging) (16). Furthermore, TMAO augments tissue factor and PAI-1, canonical SASP components, enhancing platelet hyperreactivity and thrombosis (13). Thus, increased TMAO levels may actively drive vascular senescence and thromboinflammatory remodeling. These discoveries catalyzed a broader search for additional metabolites derived from dietary protein breakdown — particularly those abundant in Western diets — that may shape cardiovascular trajectories in aging.

Aromatic amino acids, including phenylalanine, tyrosine, and tryptophan, provide the substrates from which gut microbes generate a plethora of aromatic compounds, many of which are classified as uremic toxins. A subset of these compounds act as potent signaling molecules that exacerbate CVD risk. For example, our recent studies revealed that PAA boosts the SASP by inducing redox-energy imbalance and SIRT1-dependent epigenetic remodeling, culminating in an endothelial and perivascular adipose tissue senescence phenotype and accelerated vascular aging (6). The Hazen group’s complementary metabolomic and functional studies showed that PAGln enhances platelet aggregation and thrombotic risk, while impairing cardiomyocyte contractile force (17), underscoring PAGln’s dual role in both aging and acute cardiovascular events. Beyond vascular aging, PAA and PAGln may broadly influence cardiovascular remodeling and HF pathophysiology through SIRT1-SASP modulation. As SIRT1 downregulation and SASP amplification underpin fibroblast activation, extracellular matrix remodeling, and diastolic dysfunction — key hallmarks of HF with preserved ejection fraction (1, 18) — these metabolites may exacerbate cardiomyocyte-fibroblast crosstalk, promoting myocardial stiffening and metabolic inflexibility. Targeting the metabolite/senescence axis could therefore mitigate vascular aging and disrupt multiorgan senescence circuits underpinning HF and age-related CVD. Another compelling example is histidine-derived imidazole propionate (ImP), first recognized for its contribution to impaired glucose control and type 2 diabetes (19). ImP has also been independently correlated with ASCVD and HF in humans. Recent investigations uncovered that ImP drives atherosclerosis independently of cholesterol changes, acting through both endothelial cells and macrophages (20, 21). These findings strongly support ImP’s potential as an early ASCVD biomarker, an alternative to established blood-derived biomarkers such as LDL cholesterol and C-reactive protein, that may enable identification of at-risk individuals prior to substantial plaque development.

Not all microbial metabolites exert deleterious effects. Some tryptophan catabolites, including indole-3-acetic acid (IAA) and indole-3-propionic acid (IPA), alleviate inflammation and mitigate atherosclerosis in mice and are inversely associated with cardiometabolic disease in humans. Others, such as serotonin, may conversely promote vascular inflammation (22). Short-chain fatty acids (SCFAs) are another example of a protective metabolite; for example, butyrate-producing bacteria such as *Roseburia intestinalis* remodel host metabolism and dampen systemic inflammation, thereby protecting against atherosclerosis (23). Butyrate itself restrains neutrophil extracellular trap formation, thereby mitigating abdominal aortic aneurysm development (24). These findings emphasize that CVD risk can be predicted not only by the rise of noxious microbial metabolites but also by the loss of protective ones, expanding the atlas of microbial cardiovascular signatures.

Overall, it is predicted that microbial metabolism produces over 3,000 unique chemical entities either independently or through host-microbe co-metabolism. This vast chemical repertoire suggests many more microbiota-dependent metabolites remain to be decoded for direct links with cardiovascular aging and age-associated CVD (9). Notably, microbial metabolites may exert divergent effects with age. For instance, TMAO supports osmoregulation and nitrogen balance in youth but becomes maladaptive in later life, driving vascular and immune decline. Similarly, PAA or PAGln might turn pathogenic once endothelial reserve is lost with aging. These metabolic trade-offs underscore the importance of putting the cardiovascular impact of metabolites in context with aging. Such age-dependent metabolic pleiotropy cautions that interventions targeting these metabolites could be harmful if applied uniformly across age groups. Suppressing their formation prematurely may disrupt essential microbe-host interactions governing nutrient and redox homeostasis, underscoring the need for age-specific modulation rather than global inhibition. Altogether, the findings summarized above simultaneously position microbial metabolites as an emerging class of predictive biomarkers for cardiovascular aging and emphasize prioritizing research aimed at disentangling the metabolite/CVD axis in aging populations. Looking forward, simple laboratory-based blood metabolite profiling could provide functional readouts that directly predict cardiovascular outcomes years in advance, offering a window of opportunity for lifestyle, dietary, or pharmacological interventions to preserve metabolic health and reduce late-life CVD risk.

## Gut microbiota and their metabolites as modifiable targets

Studies revealing microbial metabolites’ roles in CVD pathobiology have reframed how we consider gut microbiota, upgrading it from merely a passive biomarker to a modifiable driver of CVD. Unlike fixed determinants such as genetic or chronological age, microbiota composition and function are inherently plastic and can be reshaped by diet, pharmacological interventions, or bacterial genetic engineering. Critically, whereas many gerotherapeutics act downstream of pathology, microbiome-based strategies intervene upstream, limiting production of noxious metabolites or reinforcing protective ones.

A growing repertoire of interventions exemplifies the translational promise of targeting age-associated gut dysbiosis. Fecal microbiota transplantation (FMT) from young donors rejuvenates hematopoietic stem cells and restores immune function in aged mice (25). There are early indications of FMT’s cardiovascular benefit in small-scale clinical trials, including in patients with metabolic syndrome (26).

Building on this, next-generation probiotics, prebiotics, and postbiotics aim to enrich beneficial taxa or design strains that secrete metabolites depleted with age, such as the SCFAs acetate, propionate, and butyrate. Our recent study investigating the roles of PAA and PAGln in vascular senescence demonstrated that acetate replacement rescues this vascular senescence by restoring redox homeostasis and suppressing SASP (6).

In an intervention leveraging synthetic biology, an engineered *Escherichia coli* Nissle 1917 (EcN_TL) strain that continuously releases propionate and butyrate dampened cardiac inflammation and improved cardiac performance in a murine ischemia/reperfusion injury model (27). In parallel, deep sequencing and functional profiling have uncovered microbial pathways that can be pharmacologically targeted. One striking example is 3,3-dimethyl-1-butanol (DMB), a choline analogue that selectively inhibits microbial TMA lyases, preventing TMAO production, foam cell formation, and atherosclerotic lesion development in preclinical models (28).

More recently, selective depletion of phenylalanine-metabolizing enzymes, including α-ketoisovalerate:ferredoxin oxidoreductase (VOR), phenylpyruvate:ferredoxin oxidoreductase (PPFOR), and phenylpyruvate decarboxylase (PPDC), in specific bacteria such as *Clostridium sporogenes*, *Bacteroides thetaiotaomicron*, and *Proteus mirabilis* markedly lowered systemic levels of PAA and PAGln in gnotobiotic mice, highlighting the potential of precision microbial engineering to mitigate vascular senescence (29).

**Figure 1:**
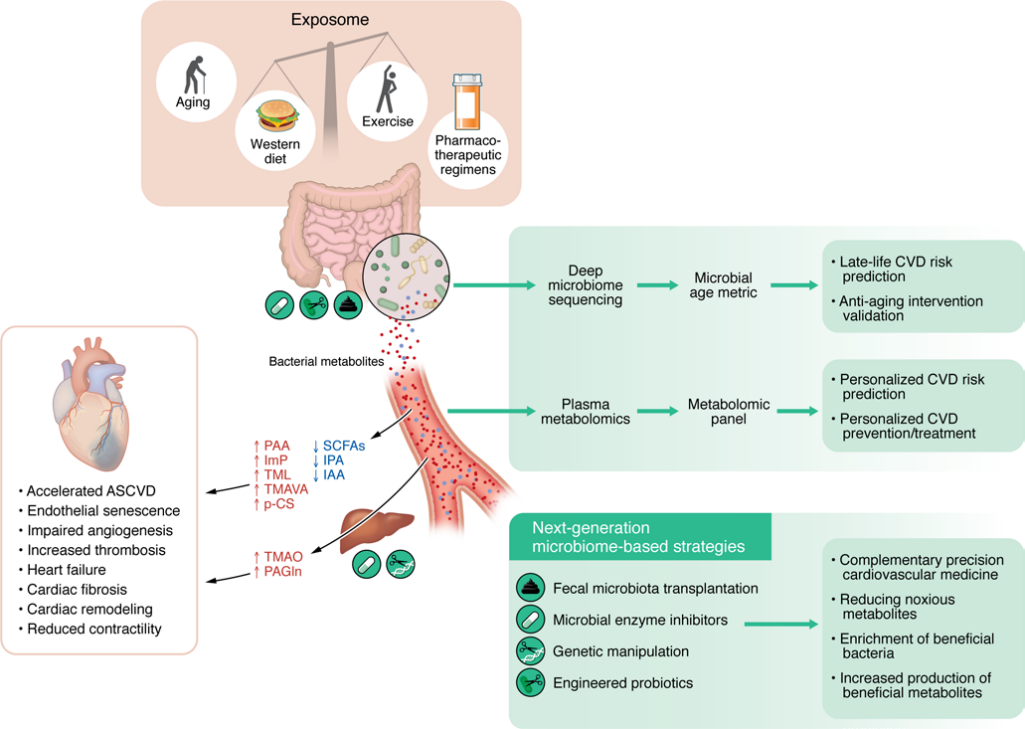
Gut microbiota as biomarker and therapeutic target in cardiovascular aging. The exposome — comprising aging, Western lifestyle patterns, and drugs — reshapes the gut microbiota, elevating noxious metabolites (e.g., TMAO, PAA, PAGln, ImP, trimethyllysine [TML], TMAVA, *p*-cresol sulfate [*p*-CS]) while depleting protective ones (e.g., SCFAs, IAA, IPA). This metabolic imbalance accelerates mechanisms underpinning adverse cardiovascular outcomes, including ASCVD and heart failure. Integrated deep fecal microbiome sequencing and plasma metabolomics, supported by advanced bioinformatics, enable the characterization of microbial age metric and metabolite panels, providing personalized late-life CVD risk prediction and establishing targeted interventions. Next-generation microbiome-based strategies — including fecal microbiota transplantation, microbial enzyme inhibitors, host-microbe genetic manipulation, and engineered probiotics — offer translational opportunities to enrich beneficial taxa, suppress CVD-associated pathways, and boost protective metabolite production, thereby advancing precision cardiovascular medicine and decelerating cardiovascular aging.

Despite encouraging progress, major translational gaps remain. The efficacy and durability of FMT depend on donor selection, transplantation stability, and safety in elderly or multimorbid patients. For next-generation probiotics and postbiotics, as well as microbial enzyme inhibitors, mechanistic clarity, dose optimization, strain-host compatibility, and off-target metabolic effects remain underexplored. The translation of microbial engineering faces regulatory and ecological hurdles, emphasizing the need for biosafety frameworks. Notably, diet-based interventions represent an immediately actionable strategy. Fiber-rich or Mediterranean diets consistently increase SCFAs while lowering TMAO and aromatic uremic toxins, thereby improving cardiometabolic profiles. Integrating nutritional, microbial, and pharmacological approaches — with age-specific precision — will be essential to move microbiome-based interventions from preclinical promise to clinical impact. Last but not least, translational efforts must take into account the impact of sex, race, and major environmental and psychosocial determinants of health — which together constitute the exposome (Figure 1) — when evaluating potential therapeutics, as these variables have been shown to substantially affect both risk and treatment efficacy in CVD.

## Challenges and future directions

Clinical translation of microbiome-based approaches is still in its early stages. A limited number of randomized trials, including GutHeart, DINAMIC, STEP-Aging, and NCT05424263, are currently evaluating cardiovascular outcomes of such interventions. Initial results from GutHeart reported no significant improvement in cardiac function with the probiotic *Saccharomyces boulardii* in patients with HF, underscoring both the promise and challenges of this field. Interindividual variability in host-microbiome interactions complicates the development of universal approaches; for example, genetic variants in hepatic flavin-containing monooxygenase 3 (*FMO3*) determine whether individuals are high or low producers of TMAO, emphasizing the necessity of tailoring personalized interventions. Moreover, ensuring the safety and durability of live microbial interventions or enzyme inhibitors is crucial, given the risk of dysbiosis, metabolic disruption, or off-target effects over extended periods. Thus, future efforts must prioritize large-scale, long-term trials that integrate stratified, mechanism-driven approaches to move beyond proof of concept and toward integration into cardiovascular therapeutics.

Looking ahead, emerging technologies promise to accelerate translational approaches. Artificial intelligence–driven pipelines can systemically screen thousands of uncharacterized metabolites and identify metabolite-mimetic drugs, such as SCFA analogues. In parallel, digital twins of host-microbe metabolism are being developed as predictive platforms for individualized responses to microbiome-based therapeutics.

## Conclusion

In summary, evidence continues to accumulate that gut microbiota is an active architect in CVD pathogenesis through its generation of bioactive metabolites that accelerate cardiovascular aging or confer resilience. Harnessing this potential of the microbiome and its metabolites requires integration of microbiome science into cardiovascular medicine through the use of microbe- and metabolite-based profiling for personalized prognostics, precision interventions, and rigorous clinical trials. With one-third of the global population affected by cardiometabolic diseases, it is our view that adopting microbiome-based strategies will be essential to addressing these conditions’ outsized impact on mortality.

## Funding support

- Swiss National Science Foundation Spark grant no. CRSK-3_229134.
- Novartis Foundation for Medical and Biological Research grant no. 21A053.
- Swiss Life Foundation grant nos. 1286, 1438, 1564, and 1565.
- Fonds zur Förderung des Akademischen Nachwuchses.
- Gebauer Stiftung (to SSSS).

## Version 1 12/01/2025

Electronic publication
